# Characterization of Enzymatic Activity of MlrB and MlrC Proteins Involved in Bacterial Degradation of Cyanotoxins Microcystins

**DOI:** 10.3390/toxins8030076

**Published:** 2016-03-16

**Authors:** Dariusz Dziga, Gabriela Zielinska, Benedykt Wladyka, Oliwia Bochenska, Anna Maksylewicz, Wojciech Strzalka, Jussi Meriluoto

**Affiliations:** 1 Department of Plant Physiology and Development, Faculty of Biochemistry, Biophysics and Biotechnology, Jagiellonian University, Gronostajowa 7, Krakow 30387, Poland; anna.maksylewicz@uj.edu.pl; 2 Department of Analytical Biochemistry, Faculty of Biochemistry, Biophysics and Biotechnology, Jagiellonian University, Gronostajowa 7, Krakow 30387, Poland; gabriela.zielinska@uj.edu.pl (G.Z.); wladykab@interia.pl (B.W.); oliwia.bochenska@gmail.com (O.B.); 3 Department of Plant Biotechnology, Faculty of Biochemistry, Biophysics and Biotechnology, Jagiellonian University, Gronostajowa 7, Krakow 30387, Poland; wojciech.strzalka@uj.edu.pl; 4 Department of Biochemistry, Faculty of Science and Engineering, Åbo Akademi University, Tykistokatu 6 A, Turku 20520, Finland; jussi.meriluoto@abo.fi

**Keywords:** microcystin, biodegradation, recombinant enzymes, biochemical pathway

## Abstract

Bacterial degradation of toxic microcystins produced by cyanobacteria is a common phenomenon. However, our understanding of the mechanisms of these processes is rudimentary. In this paper several novel discoveries regarding the action of the enzymes of the *mlr* cluster responsible for microcystin biodegradation are presented using recombinant proteins. In particular, the predicted active sites of the recombinant MlrB and MlrC were analyzed using functional enzymes and their inactive muteins. A new degradation intermediate, a hexapeptide derived from linearized microcystins by MlrC, was discovered. Furthermore, the involvement of MlrA and MlrB in further degradation of the hexapeptides was confirmed and a corrected biochemical pathway of microcystin biodegradation has been proposed.

## 1. Introduction

Cyanobacterial secondary metabolites including toxic microcystins (MCs) are subject to microbial, mainly bacterial, degradation in natural water supplies. Several MC-degraders have been identified in environmental samples [1]. However, the mechanism of MC biodegradation is poorly known and the knowledge is limited to one partially-recognized biochemical pathway [2,3] which involves three enzymes (MlrA, MlrB, and MlrC) encoded by the *mlr* gene cluster. The genome of several *Sphingomonas* sp., *Sphingophyxis* sp., but also phylogenetically distinct, bacterial species were found to contain homologues of the *mlrA* gene encoding the enzyme responsible for MC linearization, a crucial step for toxin degradation [1]. On the other hand, in such MC-degrading strains as *Arthrobacter* sp., *Brevibacterium* sp., *Rhodococcus* sp. [4], *mlr* genes were not detected. This could mean that investigated strains do not possess *mlr* homologues, but this hypothesis requires experimental verification. Regardless of the above, the mechanisms of MC-degradation in other bacterial strains may be different and not limited to the proteins of *mlr* clusters. For example, the biodegradative pathway of *Methylobacillus* sp. J10 [5] is probably based on enzymes bound to the cell wall or outside the cytoplasmic membrane. Similarly, the contribution of the cell wall-associated proteinases was demonstrated indirectly during MC-removal by probiotic bacteria, e.g., *Lactobacillus rhamnosus* [6]. Apart from proteases, some other enzymes catalyzing decarboxylation, demethylation, deamination, or dehydration reactions are possibly also involved in MC biodegradation [7,8]. However, such hypotheses are based only on the detection of degradation products, since the respective enzymes have not yet been characterized. Further studies of the mechanisms are needed to describe the alternative reaction pathways and the involved proteins.

Knowledge (on the molecular level) about the process of MC utilization by the bacteria carrying the *mlr* gene cluster is also limited. At present, neither final degradation products nor the order of their formation are known. Recombinant MC-degrading proteins may be very helpful in the investigation of the biochemical pathways and subsequent MC derivatives, and this approach has been applied in this study. The aims of this work were to heterologously express MlrC and MlrB derived from *Sphingomonas* sp., to identify and verify the putative active site of the studied enzymes and to investigate the activity of the enzymes encoded by *mlr* cluster toward MC derivatives. This approach allowed to specify the role of MlrB, MlrC, and MlrA in the bacterial utilization of microcystin heptapeptides.

## 2. Results

### 2.1. Verification of Nucleotide Sequence Coding for MlrB

The sequence of *mlrB* gene was originally provided by Bourne *et al.* [3] (AF411069—original; KR150744—revised; GenBank). Due to discrepancies of the MlrB sequence length with homologous proteins in other bacteria, as well as previous experiences with the correction of MlrA [9], we determined the sequence of the upstream *mlrB* using inverse PCR. By verification of sequencing errors, causing frameshifts resulting in truncations of N- and C-terminals of MlrB, we provided a full-length sequence which corresponds well with its homologues in *Sphingopyxis* sp. C-1 and *Sphingomonas* sp. USTB-05 [10,11]. Numeration of amino acid residues was introduced consequently, according to a postulated revised sequence of *mlrB* (Figure S1).

### 2.2. Recombinant MlrB, MlrC, and Their Mutants

**Figure 1:**
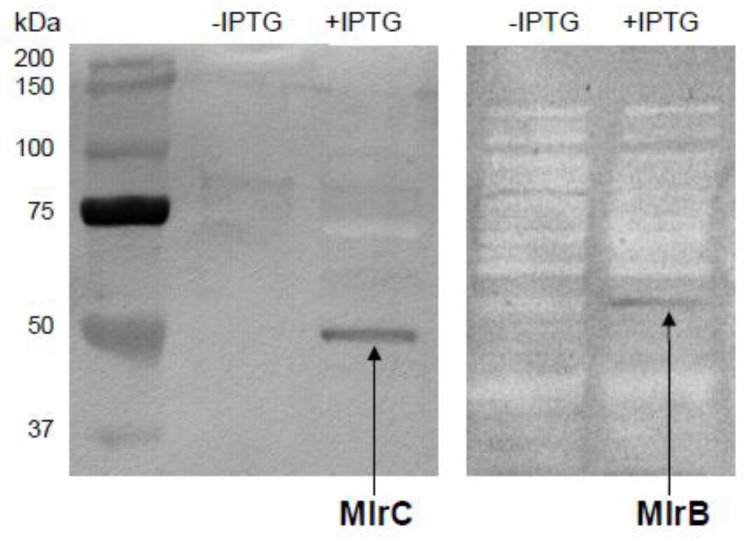
Western blot of soluble fraction of MlrC and MlrB proteins before induction (−IPTG) and after 7 h of cultivation with IPTG (+IPTG).

Based on the SDS-PAGE analysis of the *Escherichia coli* BL21(DE3) cell lysates transformed with pLATE31*mlrB* or pET21a*mlrC*, it was observed that overexpressed recombinant MlrB and MlrC were predominantly deposited in the insoluble fraction as inclusion bodies. Nevertheless, in the soluble fraction, used for protein purification in native conditions, the enzymatic activity towards linearized MCs could be detected indicating MlrB and MlrC in the supernatant. Furthermore, the addition of 15% glycerol into Luria-Bertani (LB) medium allowed to enrich the soluble fraction with both proteins (approximately 10 times higher activity in comparison with soluble fraction of proteins after cultivation without glycerol) and the presence of a significant amount of these enzymes was further confirmed by Western blotting (Figure 1). MlrB muteins MlrB_S77A_ and MlrB_K80A_ were designed based on the predicted active site [3]. Substitution of S77 and K80 residues with alanine residue abolished the enzymatic activity of MlrB, which was demonstrated using acMC-LR and acMC-RR as a substrate (Table 1). To verify the hypothesis (see Discussion for details) of the impact of three residues D167, H169, and H191 on the activity of MlrC, the muteins MlrC_D167A_, MlrC_H169A_, and MlrC_H191A_ were prepared. The lack of activity of these muteins toward acMC-LR (Table 1) confirmed that the mutated residues play the crucial role in the enzymatic activity of MlrB and MlrC.

### 2.3. MlrB and MlrC Purification

The soluble fraction of MlrB was purified on a Ni-NTA column, which allowed an efficient binding of this enzyme. After isolation, to provide sufficient ionic strength and prevent protein precipitation, purified MlrB was dialyzed against PBS buffer. The calculated recovery of MlrB activity was 30% of the initial lysate. A similar attempt was made to purify MlrC. However, despite efficient binding to Ni-NTA, only about 12% of the initial lysate activity was recovered upon elution, suggesting partial inactivation of MlrC during purification. Since nickel ions have been shown to inhibit metalloproteases through substitution of the active center ion [12], we exchanged the nickel ions for cobalt. Using a Co-NTA column we were able to obtain a highly-active MlrC preparation recovering 63% of lysate activity.

### 2.4. Activity of Recombinant MlrA, MlrB, and MlrC toward MC Derivatives

**Figure 2:**
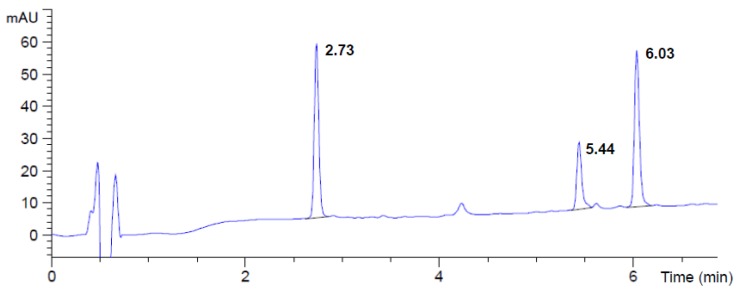
HPLC chromatogram of linearized MC-LW after degradation by MlrC. Peaks with retention times 2.73 min, 5.44 min, and 6.03 min correspond to hexaMC-LR, acMC-LR, and Adda, respectively. Monitoring at 220 nm.

**Figure 3:**
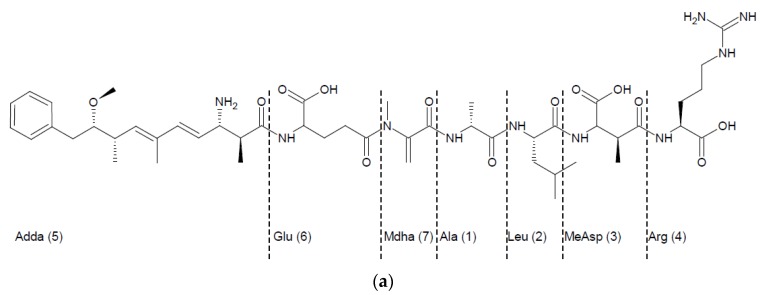

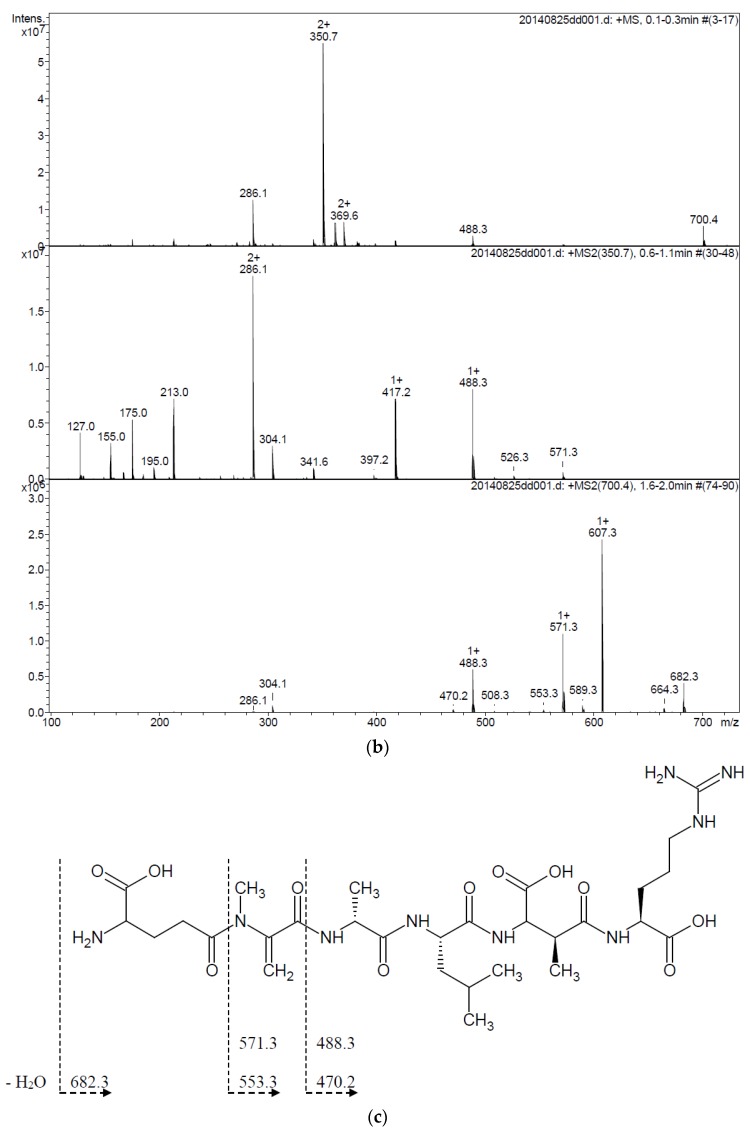
(**a**) The structure of acMC-LR with historical numbering of amino acids which is maintained in this paper; (**b**) MS and MS^2^ analysis of the hexapeptide derived from acMC-LR; after fragmentation of the *m/z* 700.4, ions at *m/z* 682.3, 607.3, 488.3 were produced; and (**c**) the structure of hexapeptide derived from acMC-LR and the proposed fragmentation pattern. The indicated fragment ions have adequate ions in the MS^2^ of at least two other hexapeptide variants (according to the differences in their initial *m*/*z* values).

Previously-unidentified products formed by hydrolysis of linearized variants of MC by MlrC were detected using a modified HPLC gradient (No 2). Increased hydrophilic conditions of separation and tracking the reaction products at 220 nm, typical for the peptide bond chromophore allowed to detect the molecules without the Adda-conjugated diene chromophore. Examples of HPLC chromatograms are shown in Figure 2 and Figure S2. In addition to the peak corresponding to Adda (6.03 min), one new product with different retention time (depending on the acMC variants) was detected for each substrate used (Table 2). The MS analysis (indicating the *m/z* peaks 700.4 and 350.7) and MS/MS fragmentation pattern of acMC-LR derivative (Figure 3a) allowed recognition of this product as a hexapeptide Glu-Mdha-Ala-Leu-MeAsp-Arg, named hexaMC-LR (Figure 3b,c). The fragments with *m/z* 571.3, 553.3, and 488.3 were identical with those reported by Imanishi, *et al.* (2005) [13] and proved that the isolated product consists of at least five aa (Mdha-Ala-Leu-MeAsp-Arg), whereas the fragment with *m*/*z* 682.3 is postulated to be Glu-Mdha-Ala-Leu-MeAsp-Arg with water loss. Depending on the acMC variant used (Table 2), hexapeptides produced from other acMC variants had *m/z* values as expected based on the calculation of their molecular masses and their fragmentation patterns also corresponded to those obtained by the fragmentation of the hexaMC-LR derivative (Figure S3). Based on this analysis, the cleavage site (peptide bond between Adda-Glu) of the linearized variants was recognized.

**Figure 4:**
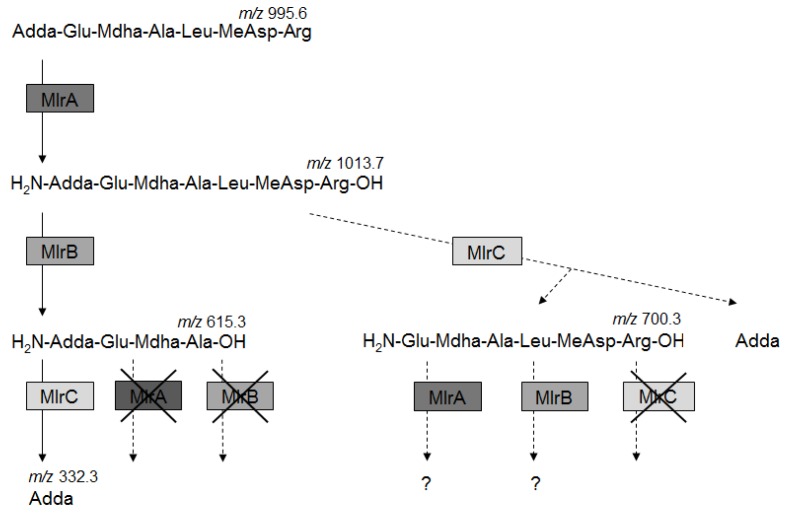
MC-LR biodegradation pathway present in bacterial strains possessing the *mlr* cluster. The same scheme of degradation of other MC variants has been confirmed. Dashed arrows indicate the data reported by the authors.

In the next step, the activity of MlrA, MlrB, and MlrC towards purified hexapeptides (hexaMC-LR and hexaMC-RR) was tested. It was documented that the hexapeptides were not further degraded by MlrC. On the other hand, a careful analysis of the hexapeptide concentration after incubation with MlrA and MlrB documented that these products are hydrolyzed by these two enzymes (Table 1). This observation was supported by assays with (a) known inhibitors of MlrA or MlrB and (b) mutated MlrA [9] or MlrB. In the presence of phenanthroline (MlrA activity assay) and PMSF (MlrB activity assay) and during incubation with muteins (MlrA_H260A_ and MlrB_S77A_), the concentration of tested hexapeptides did not change. In addition to above described analysis a tetrapeptide, another well-known degradation product of MlrB, was purified and tested as a possible substrate of MlrA, MlrB, or MlrC. It was observed that MlrA and MlrB were not active toward this product, while MlrC released Adda from the tetrapeptide. Finally, both positive and negative results of enzymatic assays allowed to obtain new information, which may be summarized as follows: (1) MlrB is able to hydrolyze linearized heptapeptides derived from different MC variants independently on the amino acid residue at position 2 (Leu in MC-LR, MC-LF, MC-LW, MC-LY, Arg in MC-RR and Tyr in MC-YR); (2) linearized variants of several most common MCs are hydrolyzed by MlrC within the peptide bond between Adda and Glu, resulting in Adda and hexapeptides as products; (3) the hexapeptides are not further degraded by MlrC; but (4) are decomposed by MlrA and MlrB, however products have not been detected; and (5) the tetrapeptide is not further degraded either by MlrA or MlrB. Based on these findings we have introduced several corrections to the scheme of MC biodegradation (Figure 4).

## 3. Discussion

### 3.1. Recombinant MlrB and MlrC—Verification of Sequence, Construction, and Analysis of Muteins, Purification

Previous studies of recombinant MlrA [9] confirmed that the active site of this protein is located within H_260_AIHNE_265_. The study of MlrB revealed that it is a serine protease [2] which has a strong sequence similarity to members of the penicillin-recognizing enzyme family, with the conservative sequence Ser-Xaa-Xaa-Lys, and to a number of β-lactamases [3,14]. However, such activity of MlrB has not been documented to date. In the present study we have verified, experimentally, the predicted active center of MlrB. The substitution of Ser (S_77_) and Lys (K_80_) with Ala residue completely abolished the activity of the enzyme against linearized MCs. The studies of MlrC metalloprotease showed that EDTA and o-phenantroline abolished enzymatic activity of this enzyme [2]. However, no conservative motifs typical for metallopeptidases were found in the sequence of this protein. The sequence comparison, performed using BLASTP algorithm (BLOSUM 62 matrix) indicated that MlrC of *Sphingomonas* sp. ACM-3962 shows 24% identity and 40% sequence similarity with respect to MlrC-like metallopeptidase (accession number in UniProt: Q11B79) derived from *Mesorhisobium* sp*.* BNC1, with known crystallographic structure (Protein Data Bank database identifier: 3IUU). Structural studies demonstrated that in a MlrC-like protein D_138_, H_140_, and H_162_ amino acid residues bind zinc ions [15]. Interestingly, in MlrC of *Sphingomonas* sp*.* ACM-3962 the respective residues are present at positions D_167_, H_169_, and H_191_. In order to verify the impact of these residues on the activity of MlrC, three independent muteins MlrC_D167A_, MlrC_H169A_, and MlrC_H191A_ were prepared. None of the tested MlrC mutants exhibited an activity against linearized MCs and tetrapeptides, which confirmed that the mutated residues play a crucial role in the enzymatic activity of MlrC. Moreover the requirement of a divalent metal ion (presumably Zn^2+^) in the active site of MlrC has been indirectly confirmed based on the previous report on the MlrC-like protein [15]. The analysis of MlrC purified using a Ni-NTA column demonstrated that the majority of the initial enzyme activity was lost. In contrast to this result, substantial improvement of enzyme activity was observed when the resin with immobilized cobalt ions was used instead of Ni-NTA. A similar effect was observed during the purification of a Zn-dependent metalloprotease, lysostaphin; application of the resin with immobilized cobalt ions allowed for a substantial improvement of IMAC-based purification [12].

### 3.2. MCs Degradation Pathway

The first well-confirmed scheme of MCs degradation [2] assumed a sequential degradation of these toxins by MlrA, MlrB, and MlrC enzymes encoded by genes of *mlr* cluster [3]. Three well-confirmed intermediates of such degradation have been detected: acMC-LR, a tetrapeptide [2] (Figure 3b), and Adda [16]. General knowledge concerning the activity of Mlr enzymes against MCs was as follows: MlrA expresses activity toward cyclic MCs and nodularin (NOD) and it hydrolyzes the peptide bond between Adda (position 5) and the amino acid residue at position 4, regardless of the MC variant, causing linearization of molecules. MlrB is able to catalyze the hydrolysis of linearized MCs by cutting the peptide bond between Ala (position 1) and a variable amino acid residue at position 2; however, the resulting tetrapeptides originating only from MC variants with Leu at position 2 have been reported to date [2,14,16,17]. MlrC releases Adda by hydrolysis of a peptide bond between Adda and Glu both in the linearized heptapetide [18] and the tetrapeptide. Apart from Adda, several other MC degradation products were also detected [19], mainly tri- and dipeptides. However the order of their formation was not fully clarified and the involvement of particular enzymes was not addressed in that study. Finally, the general opinion was that MCs are linearized by a very specific MlrA enzyme, and then the heptapeptides are completely degraded by MlrB and MlrC to single amino acids. In the current study we are presenting more comprehensive data concerning the role of enzymes encoded by the *mlr* gene cluster in further utilization of linearized MCs.

Among different MC-LR degradation products hexapeptides have never been predicted nor detected and our findings constitute the first report of the formation of such products as a result of MlrC activity. In wild strains, these intermediates are probably hydrolyzed immediately by other enzymes. It may be the reason why hexapeptides have never been detected when MCs biodegradation was investigated using wild bacterial strains. The use of single enzymes allowed us to recognize these intermediates and to verify activity of studied proteins.

The documented activity of MlrC sheds new light on the specificity of this enzyme. Previously [18], we found Adda as the only degradation product of linearized heptapeptide hydrolysis and we suggested that MlrC is less specific and can hydrolyze other bonds of linearized MC derivatives, producing small peptides and amino acids. Current research strongly supports the hypothesis that MlrC can hydrolyze only one peptide bond within acyclic MCs (between Adda and Glu) which was assumed from the fact that the hexapeptides (regardless MC variant they came from) are not further degraded by MlrC.

On the other hand, the hexpeptides are degraded both by MlrA and MlrB. The hydrolysis products were not detected. Nevertheless we postulate (based on the analysis of the degradation of several acyclic MC variants) that MlrB may be active toward Ala-X bond (where X denotes a variable amino acid at position 2 in different variants of MC), which results in the formation of two tripeptides (Glu-Mdha-Ala and X-(Me)Asp-Z). This also implies that the tripeptide Adda-Glu-Mdha documented by Hashimoto, *et al.* (2009) [19] is formed neither by MlrB nor MlrC. Most surprising, however, is the finding of MlrA activity against the hexapeptide (regardless of the MC variant). To date, only cyclic toxins have been proved to be a substrate for this enzyme. Among 50 tested fluorogenic and chromogenic synthetic substrates, none were hydrolyzed by MlrA [9]; hence, this protease was suggested to be very specific. The current results shed new light on the role of MlrA in complete utilization of MCs.

Adda is known to determine the protein phosphatase (1 and 2A) inhibition capacity of MCs and related pentapeptide NOD [20]. It has been demonstrated that this amino acid induces the expression of *mlrA* and *mlrB* genes [21] and plays an important role in the bacterial decomposition of MCs. Moreover, the authors concluded that Adda is a key signaling molecule involved in cell-to-cell communication. Our results provide new arguments supporting this hypothesis. We indicated that both MlrA and MlrC are active toward MCs, or their fragments as long as they contain Adda. Moreover, MlrC cleaves only the peptide bond between Adda and Glu. However, hydrolysis is possible only after previous linearization of heptapeptides by MlrA. The latter recognizes the peptide bond at the N-terminal site of Adda independently on the variable amino acid residue at position 4. On the other hand, other peptide bonds of hexapeptides may be also hydrolyzed by this enzyme, but only when Adda is removed by MlrC; however, details of this phenomenon are currently unknown.

Some of the above-mentioned proposals require experimental confirmation but our hypothesis is that the final products of MlrB and MlrC activity toward linearized MCs are Adda, Glu-Mdha-Ala, and X-MeAsp-Z. It leaves open the question whether these two tripeptides are utilized by MlrA or/and other enzymes, not encoded in the *mlr* cluster. To answer the question, it is necessary to detect and purify new degradation products which can be subsequently tested to determine whether they are susceptible to Mlr enzymes. If not, it would open the area of investigation of the other enzymes of *Sphingomonas* sp. involved in complete utilization of MCs.

Safety plans of World Health Organization regarding treatment strategies for the mitigation of cyanobacteria and their metabolites assume the development of many options of water purification which also include biological treatment. Academic research of the mechanism of bacterial degradation of MCs allow to deepen our knowledge in this area. Such advanced research conducted simultaneously with environmental studies help in better understanding the phenomenon of MCs’ biodegradation and should result in more relevant application studies.

## 4. Materials and Methods

### 4.1. Chemicals and Bacterial Strains 

Trifluoroacetic acid (TFA) was obtained from Sigma (St Louis, MO, USA); C18 Purospher column and acetonitrile (ACN) were obtained from Merck (Darmstadt, Germany). pTZ57R/T cloning vector, expression vector pLATE31, Phusion High-Fidelity DNA Polymerase, T4 DNA ligase, NdeI and NotI were obtained from Thermo Fisher Scientific (Waltham, MA, USA); *E. coli* BL21(DE3) and expression vector pET21a from Novagen (Darmstadt, Germany). Monoclonal antibodies and peroxidase-conjugated secondary antibodies were from Sigma (St Louis, MO, USA). Linearized MC variants were produced from cyclic forms by the method described earlier [9]. MC-LR and dmMC-LR were extracted and purified from a culture of *Microcystis aeruginosa* PCC 7813 strain (the Pasteur Institute, Paris, France) [22]. MC-LW, MC-LF (extracted from *M. aeruginosa* PCC7820), MC-RR, MC-LY, and MC-YR (*Microcystis* NIES 107) were HPLC purified as described by Meriluoto and Spoof [23]. MlrA used in this study was expressed in *E. coli* BL21 pET21a*mlrA* and purified as described previously [9]. All acyclic MCs were produced by linearization of cyclic MC variants by MlrA, whereas tetrapeptide was produced from acMC-LR by MlrC. These products were purified by HPLC followed by MS analyses (description in Section 4.7).

### 4.2. Verification of MlrB Coding Sequence

To determine DNA sequence upstream *mlrB* coding sequence inverse PCR method has been used. Briefly, genomic DNA from *Sphingomonas* sp. ACM-3692 has been digested with NcoI and subsequently purified with CleanUp (A & A Biotechnology, Gdynia, Poland). The digested DNA was circularized with T4 DNA ligase. Aliquots of the ligation mixture were used as a template in inverse PCR with primers InvBF and InvBR pointing outwards (Table 3).

### 4.3. Construction of Recombinant Plasmids, Including Mlrb and Mlrc and Their Mutants

MlrB and mlrC ORFs were amplified using *Sphingomonas* sp. ACM-3692 genomic DNA as a template. After amplification with mlrBF and mlrBR primers (Table 3), mlrB was inserted into pLATE31 vector using an aLICator Ligation Independent Cloning and Expression System (Thermo Fisher Scientific, Waltham, MA, USA) according to the manufacturer’s instruction. The resulting plasmid (pLATE31mlrB) encodes full-length MlrB (541 amino acid residues) with a six-histidine tag added to *C*-terminus. The amplified *mlrC* fragment was inserted into a pTZ57R/T cloning vector, cut out using NdeI and NotI restriction enzymes, and inserted into the expression vector pET21a. The resulting plasmid pET21amlrC encodes full length MlrC (507 amino acids) with a His tag on its C-terminal. Particular muteins were prepared based on the initial constructs pLATE31mlrB and pET21amlrC by site-directed mutagenesis method. Codons selected for the mutagenesis were replaced with codon for alanine introduced with primers (Table 4). The PCR was carried out with a primer pair introducing a mutation (Table 3) and Phusion polymerase.

### 4.4. Expression of Recombinant MlrB and MlrC

Competent *E. coli* BL21(DE3) cells were transformed with pET21amlrC, pET21amlrC_D167A_, pET21amlrC_H169A_, pET21amlrC_H191A_, pLATE31mlrB, pLATE31mlrB_S77A_, or pLATE31mlrB_K80A_ plasmids and plated on LB agar plates containing ampicillin (100 μg·mL^−1^). As a negative control, *E. coli* BL21(DE3) transformed with pET21a or pLATE31 were used. A single colony was inoculated into liquid LB medium supplemented with 15% glycerol and with ampicillin (100 μg·mL^−1^), and grown at 37 °C with vigorous shaking. When the culture reached OD_600_ = 0.8 the expression of cloned genes was induced by the addition of isopropyl β-d-1-thiogalactopyranoside (IPTG, from Lab Empire S.C., Rzeszow, Poland) to a final concentration of 1 mM. Next, the temperature was reduced to 22 °C and the cells overexpressing MlrB and MlrC were grown for 20 h. Subsequently, the bacteria were centrifuged and the pellet was stored at −20 °C until use.

### 4.5. Purification of Recombinant MlrB and MlrC

The purification based on Ni-NTA (Qiagen, Hilden, Germany) was applied using conditions similar to these recommended by the supplier. Upon centrifugation, the *E. coli* BL21(DE3) cells were suspended in a lysis buffer (50 mM NaH_2_PO_4_, 300 mM NaCl, pH 8.0), sonicated, followed by centrifugation. The supernatants containing recombinant MlrB and MlrC (or lysates deriving from cells carrying empty plasmids as negative control) were loaded into the Ni-NTA and Co-NTA column, respectively. Co-NTA was prepared from Ni-NTA by removing nickel ions (washing with 100 mM EDTA, pH 8.0) and loading cobalt ions (washing the column with 100 mM CoCl_2_). After loading, the columns were washed with lysis buffer and then recombinant proteins were eluted with elution buffer containing 250 mM imidazole. Protein-containing fractions were immediately dialyzed against PBS, pH 7.0 (MlrB) or 50 mM phosphate buffer, pH 7.0 (MlrC). SDS-PAGE [24] was used to analyze protein expression (soluble and insoluble fractions) and the purification process. Western blot was used to confirm protein expression in soluble fractions. Briefly, upon electrophoresis proteins were electrotransfered onto polyvinylidene fluoride (PVDF) membrane with 10 mM 3-(cyclohexylamino)-1-propanesulfonic acid pH 11.0. The membrane was incubated with anti-His tag monoclonal antibodies. After incubation with the peroxidase-conjugated secondary antibodies the blot was developed using BCIP/NBT as a substrate.

### 4.6. Activity Assays

The *E. coli* pellet was suspended in 50 mM sodium phosphate pH 7.0 (1/20 volume of bacterial culture) and the cells were disrupted by sonication. Acyclic MC-LR (acMC-LR) and linearized variants originating from other MCs were used to test the activity of the MlrA, MlrB, MlrC, and their mutated forms. Ten μL of the *E. coli* lysate or purified enzyme in serial dilution was added to 90 μL of acMC-LR or other MC derivative (1.0 μg·mL^−1^) in 50 mM phosphate buffer, pH 7.0. The samples were incubated at 20 °C for 1 h and the reaction was stopped by addition of 10 μL of 1% TFA. The production of tetrapeptide and Adda from acMC-LR by MlrB and MlrC, respectively, was monitored by HPLC. MlrA, MlrB, and MlrC activity towards different MC derivatives (hexapeptides and tetrapeptide) was performed as described above with some modification of substrate concentration and time of reaction, depending on the experiment. To confirm the MlrA and MlrB activity towards hexapeptides derived from acMC-LR and acMC-RR, three types of negative control were used: (1) mutated enzymatically-inactive MlrA [9], MlrB, and MlrC muteins, (2) crude extracts, and (3) samples purified from total protein extract of *E. coli* cells transformed previously with empty plasmids. Additionally, control assays were performed in the presence of o-phenantroline (inhibitor of metallopeptidases) or phenylmethanesulfonyl fluoride (PMSF, inhibitor of serine proteases) with a final concentration of 20 mM and 4 mM, respectively. The lack of activity was confirmed by the lack of changes in the substrate concentration.

### 4.7. HPLC and Mass Spectrometry

HPLC analyses, including the identification of the degradation products of acMCs, were performed using an Agilent 1220 Infinity Gradient DAD LC System (Aglient Technologies, Santa Clara, CA, USA) including gradient pump with an integrated degassing unit, autosampler, column oven, and diode array detector. MC degradation products were separated and quantified using a Purospher STAR RP-18 endcapped column (55 mm × 4 mm, 3 μm particles). The mobile phase consisted of a gradient of 0.05% aqueous TFA (solvent A) and 0.05% TFA in acetonitrile (solvent B). Most of the assays were performed with the following linear gradient program (Number 1): 0 min 25% B, 5 min 70% B, 6 min 70% B, and 6.1 min 25% B. Additionally, to detect the hexapeptides, the following gradient program (Number 2) was applied: 0 min 5% B, 8 min 70% B, 9 min 70% B, and 9.1 min 5% B. Fractions obtained from HPLC separations were evaporated to dryness and dissolved in 30% methanol with 0.1% HCOOH. Samples were analyzed using a HCTultra ETDII mass spectrometer (Bruker, Bremen, Germany). The system was operated with a syringe pump (KD Scientific, Holliston, MA, USA) at a flow rate of 3 μL·min^−1^ and direct injection of samples to an electrospray ionization (ESI) ion source. The ESI was operated in the positive ion mode, at the capillary voltage of 3.5 kV, nebulizer pressure of 10 psi, drying gas flow of 5 L·min^−1^ and ion source temperature of 300 °C. The ion trap (IT) analyzer performed both MS and MS^2^ (tandem mass spectrometry) analyses. Peptide identification was performed manually using DataAnalysis™ 4.0 software (Bruker, Bremen, Germany).

## Figures and Tables

**Table 1:** The summarized results of the activity of Mlr enzymes and their muteins toward the derivatives of different MC variants.

MC Derivatives	MlrA	MlrA_H260A_	MlrB	MlrB_S77A_	MlrC	MlrC_H169A_	* Empty Plasmids
acMC-LR	−	−	+	−	+	−	−
acdmMC-LR	−	−	*n.a.*	−	+	−	−
acMC-LF	−	−	*n.a.*	−	+	−	−
acMC-LW	−	−	*n.a.*	−	+	−	−
acMC-LY	−	−	*n.a.*	−	+	−	−
acMC-RR	−	−	+	−	+	−	−
acMC-YR	−	−	*n.a.*	−	+	−	−
hexaMC-LR	+	−	+	−	−	−	−
hexaMC-RR	+	−	+	−	−	−	−
tetrapeptide	−	−	−	−	+	−	−

*n.a.*—not analyzed in this study; * crude extracts and samples purified from total protein extract of *E. coli* cells transformed previously with empty plasmids.

**Table 2:** Retention times and *m/z* values of linearized MC variants and their derivatives after hydrolysis by MlrC. Asterisks indicate doubly-protonated ions.

Linearized Variants of Different MC	MC-LR	dmMC-LR	MC-LW	MC-LF	MC-RR	MC-YR	MC-LY
*m/z* of heptapeptides	1013.7	999.7	1043.6	1004.6	1056.5 528.9 *	1063.7	1020.6
retention time (min)	5.4	5.3	6.7	6.6	4.6	5.1	6.1
*m/z* of hexapeptides	700.4 350.7 *	686.4 343.7 *	730.4	691.4	372.3 *	750.4 375.7 *	707.4
retention time (min)	2.7	2.7	4.7	4.7	1.7	2.6	3.8

Second and third lines indicate *m/z* values and retention times of linear derivatives of MC, respectively. Fourth and fifth lines indicate *m/z* values and retention times of hexapeptides derived from the hydrolysis of linear MCs, respectively.

**Table 3:** Primers used in the construction of recombinant plasmids and mutagenesis. Artificial sequences facilitating cloning to pLATE and pET21 are italized, codon introducing mutations are bolded. InvBF and InvBR primers were used in inverse PCR.

Primer Name	Sequence (5′ to 3′)	Amplified Fragment Length (nt)
InvBF	CAAAGCCGCCCTGAAAAAGAAC	-
InvBR	TATGCCGGTGGATTGTTCGTC	
mlrBF	*AGAAGGAGATATAACT*ATGACTGCAACAAAGCTTTTCCTGGCG	1660
mlrBR	*GTGGTGGTGATGGTGATGGCC*TCGAAGCCGCCTGAACACTATCCCGTTCAG	
mlrBS77AF	CTTCGAGTTGGCG**GCA**ACATCGAAGC	~6124 *
mlrBS77AR	GCTTCGATGT**TGC**CGCCAACTCGAAG	
mlrBK80AF	CGTCAACATCG**GCG**CAGTTTACAGC	~6124 *
mlrBK80AR	GCTGTAAACTG**CGC**CGATGTTGACG	
mlrCF	*GTTCCATATG*CTTGATCGTCGAACATTG	1577
mlrCR	*GAAAGCGGCCGCG*ACAGGCTCGAATGGCCAC	
mlrCD167AF	GGGGCCGAACTT**GCT**CTTCACGCTCAC	6947 *
mlrCD167AR	GTGAGCGTGAAG**AGC**AAGTTCGGCCCC	
mlrCH169AF	GAACTTGATCTT**GCC**GCTCACTTGTCG	6947 *
mlrCH169AR	CGACAAGTGAGC**GGC**AAGATCAAGTTC	
mlrCH191AF	CAAGTACTATCCG**GCT**ATCGACTACGTC	6947 *
mlrCH191AR	GACGTAGTCGAT**AGC**CGGATAGTACTTG	

* primers were used in site directed mutagenesis method, the whole plasmid containing insert with substituted codon was amplified.

**Table 4:** Amino acid residues and respective codons exchanged in MlrB and MlrC mutant studies.

Protein	Substituted		Introduced	
	Residue	Codon	Residue	Codon
MlrB	S_77_	TCA	A_77_	GCA
	K_80_	AAG	A_80_	GCG
MlrC	H_167_	GAT	A_167_	GCT
	D_169_	CAC	A_169_	GCC
	H_191_	CAT	H_191_	GCT

## Supplementary Materials

The following are available online at www.mdpi.com/2072-6651/8/3/76/s1.

## Abbreviations

The following abbreviations are used in this manuscript:

acMCs	(linearized) acyclic microcystins
Adda	3-amino-9-methoxy-2,6,8-trimethyl-10-phenyl-deca-4,6-dienoic acid
BLAST	Basic Local Alignment Search Tool
Co-NTA	Ni-NTA column with nickel ions replaced with cobalt ions
hexaMCs	hexapeptides produced from acyclic MCs
HPLC	high performance liquid chromatography
IPTG	isopropyl β-d-1-thiogalactopyranoside
*m/z*	mass to charge ratio
MC	microcystin
Mdha	methyldehydroalanine
NOD	nodularin
ORFs	open reading frames
PCR	polymerase chain reaction
PMSF	phenylmethanesulfonyl fluoride
