# Supplementary material for: Characterization of Enzymatic Activity of MlrB and MlrC Proteins Involved in Bacterial Degradation of Cyanotoxins Microcystins
Source: Toxins (Basel). 2016 Mar 16;8(3):76. doi: 10.3390/toxins8030076 (PMC4810221; doi:10.3390/toxins8030076)
Supplement: Supplementary file 1 [file toxins-08-00076-s001.pdf]

Supplementary Materials: Characterization of Enzymatic Activity of MlrB and MlrC Proteins Involved in Bacterial Degradation of Cyanotoxins Microcystins

Dariusz Dziga, Gabriela Zielinska, Benedykt Wladyka, Oliwia Bochenska, Anna Maksylewicz, Wojciech Strzalka and Jussi Meriluoto

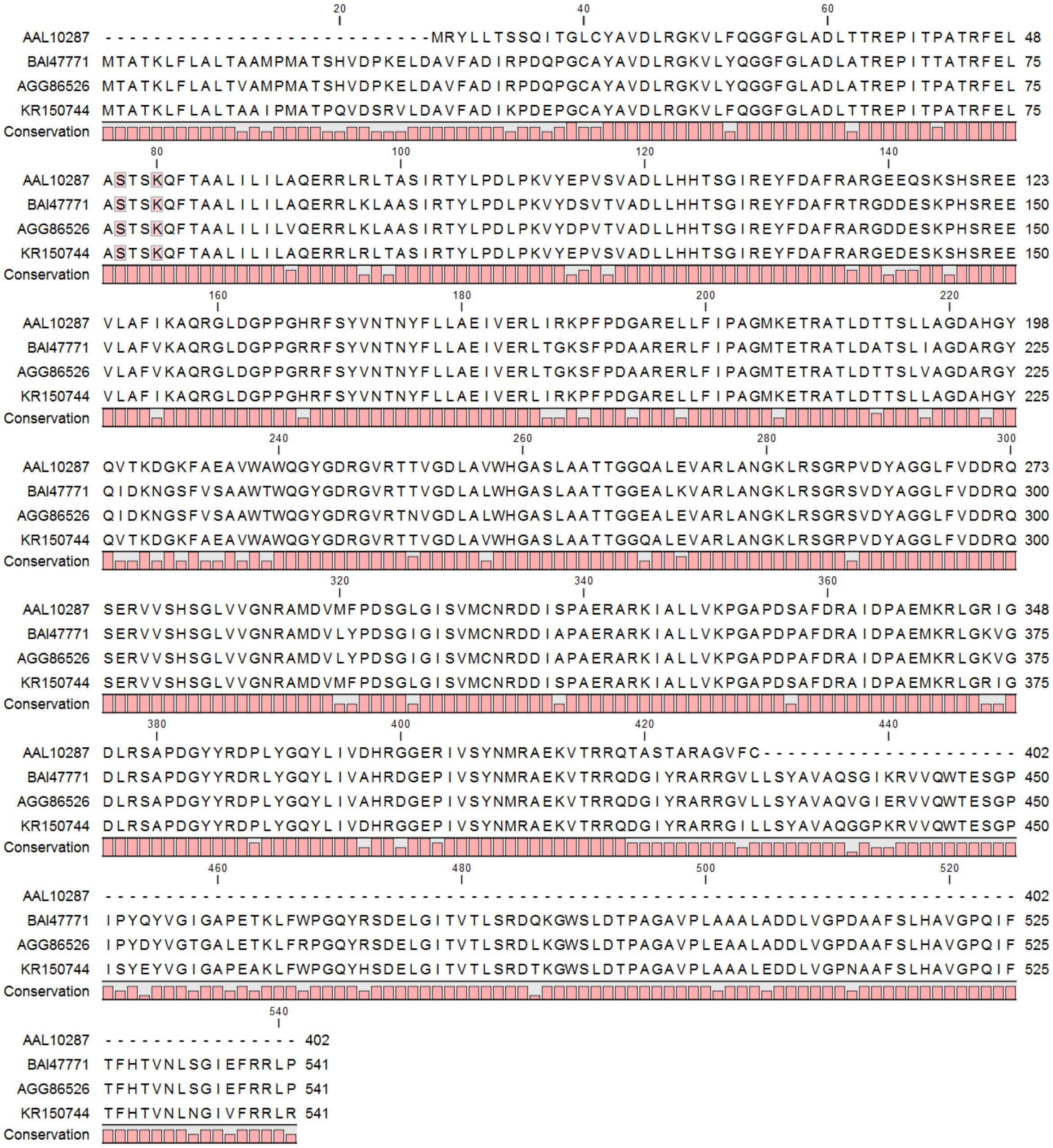

Figure S1. Alignment of MlrB protein amino acid sequences from bacterial strains of *Sphingomonaceae* family (AAL10287—*Sphingomonas* sp. ACM-3962 (Bourne, 2001), BAI47771—*Sphingopyxis* sp. C-1, AGG86526—*Sphingomonas* sp. USTB-05, KR150744—*Sphingomonas* sp. ACM-3962) verified in the present study.

acLR_negative control

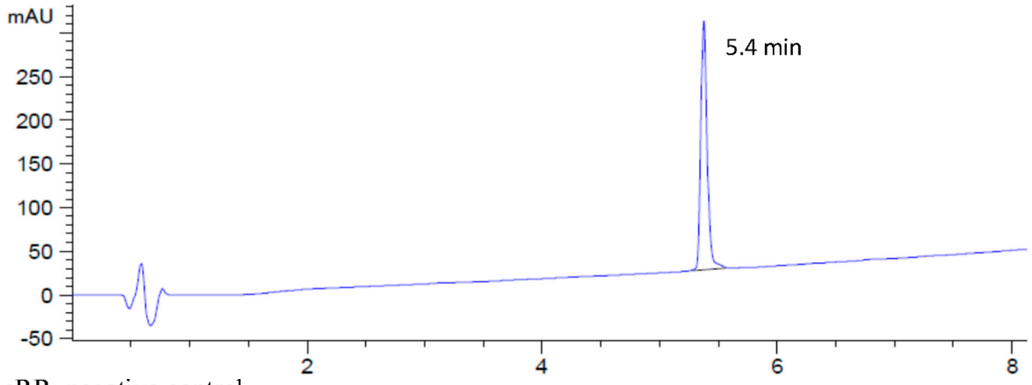

acRR_negative control

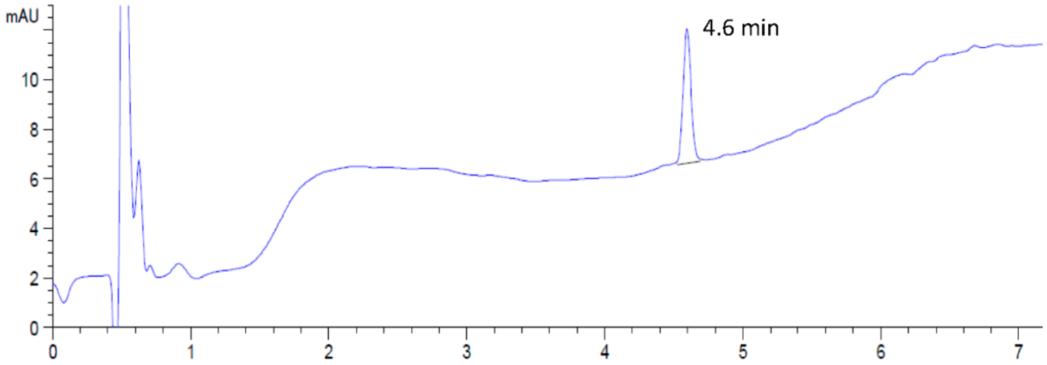

acRR_MlrC

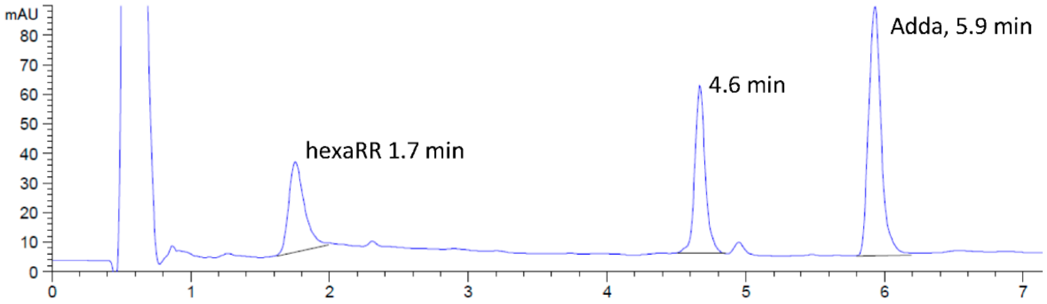

Figure S2. *Cont.*

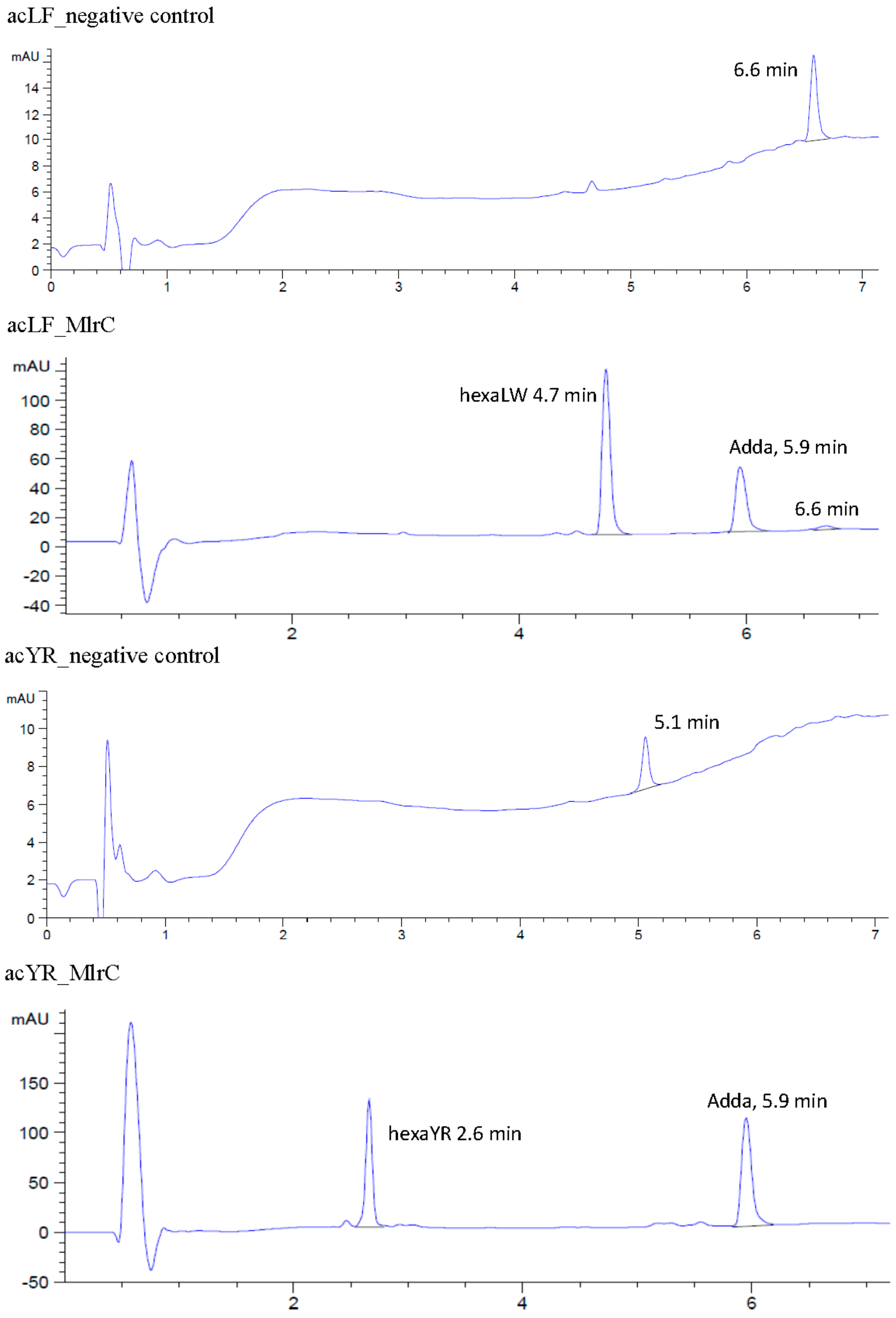

Figure S2. The examples of chromatograms indicating the formation of hexapeptides from different linearized MC variants by MlrC and the lack of degradation of linearized variants in the presence of extract of *E. coli* with empty plasmid pET21a (negative controls).

hexaMC-LF:

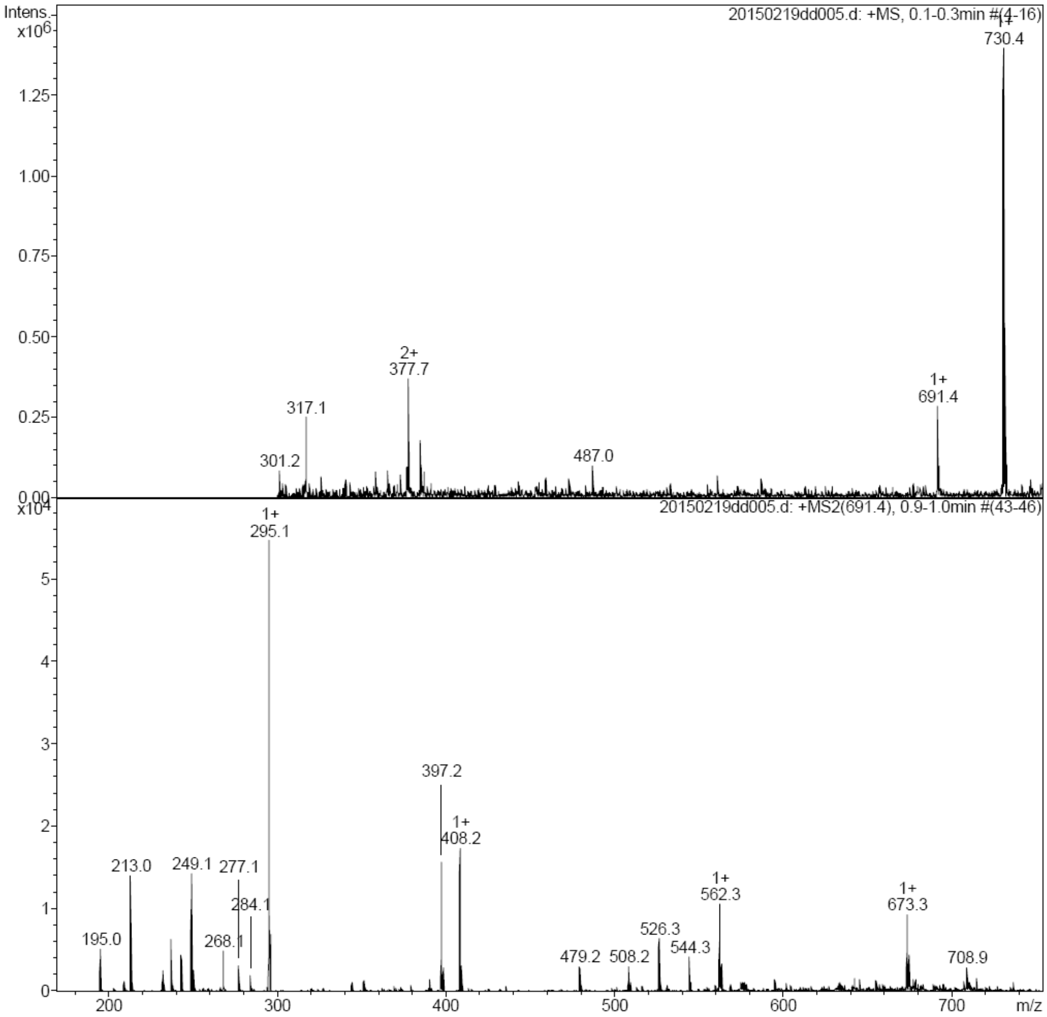

Figure S3. *Cont.*

hexaMC-LW:

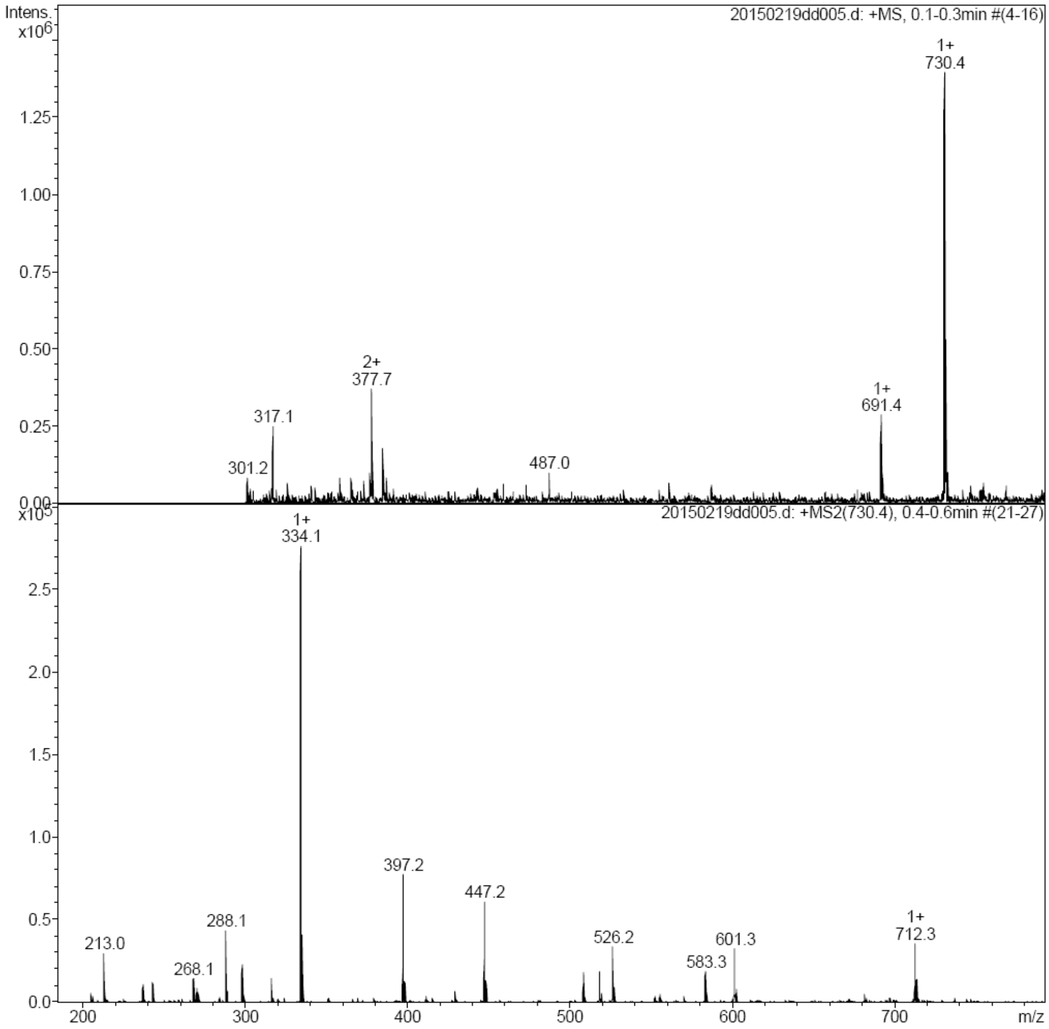

Figure S3. *Cont.*

hexaMC-LY:

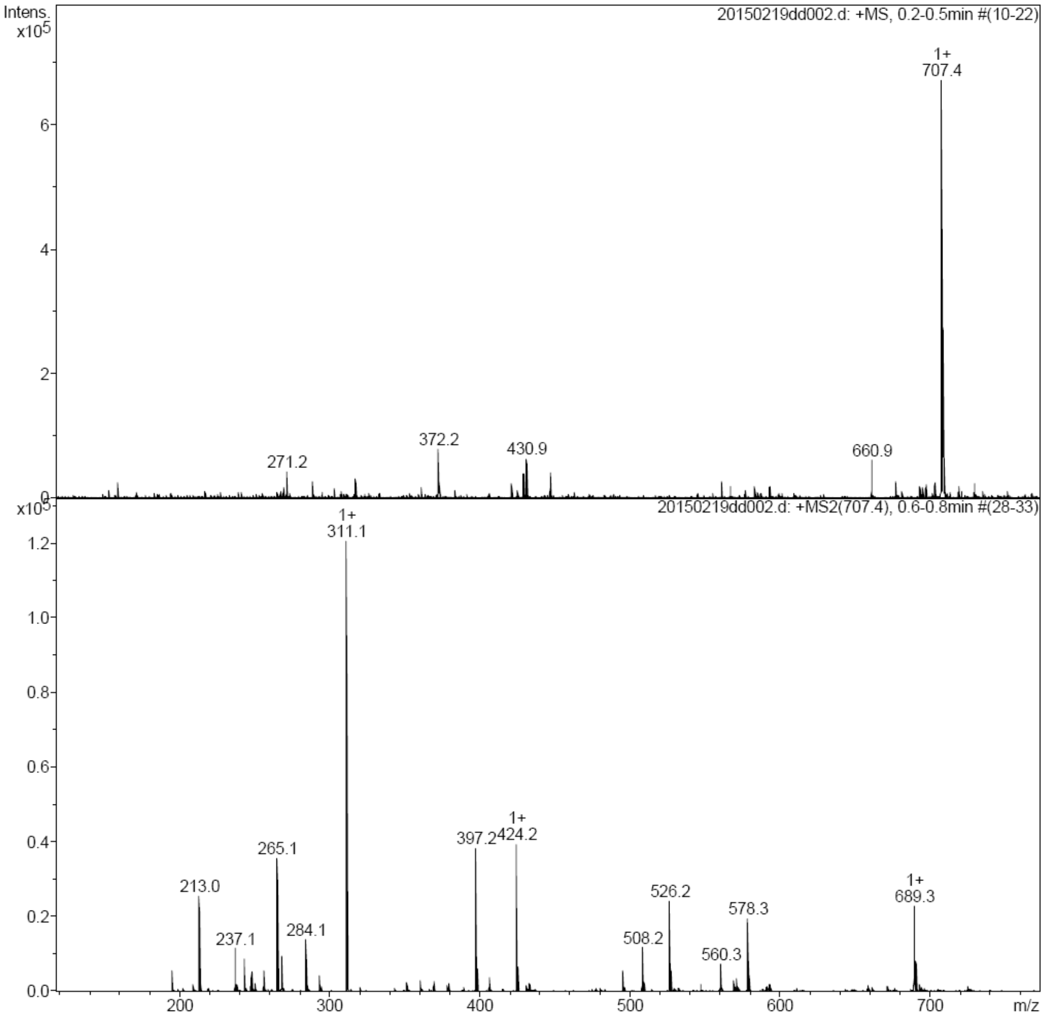

Figure S3. *Cont.*

hexaMC-RR:

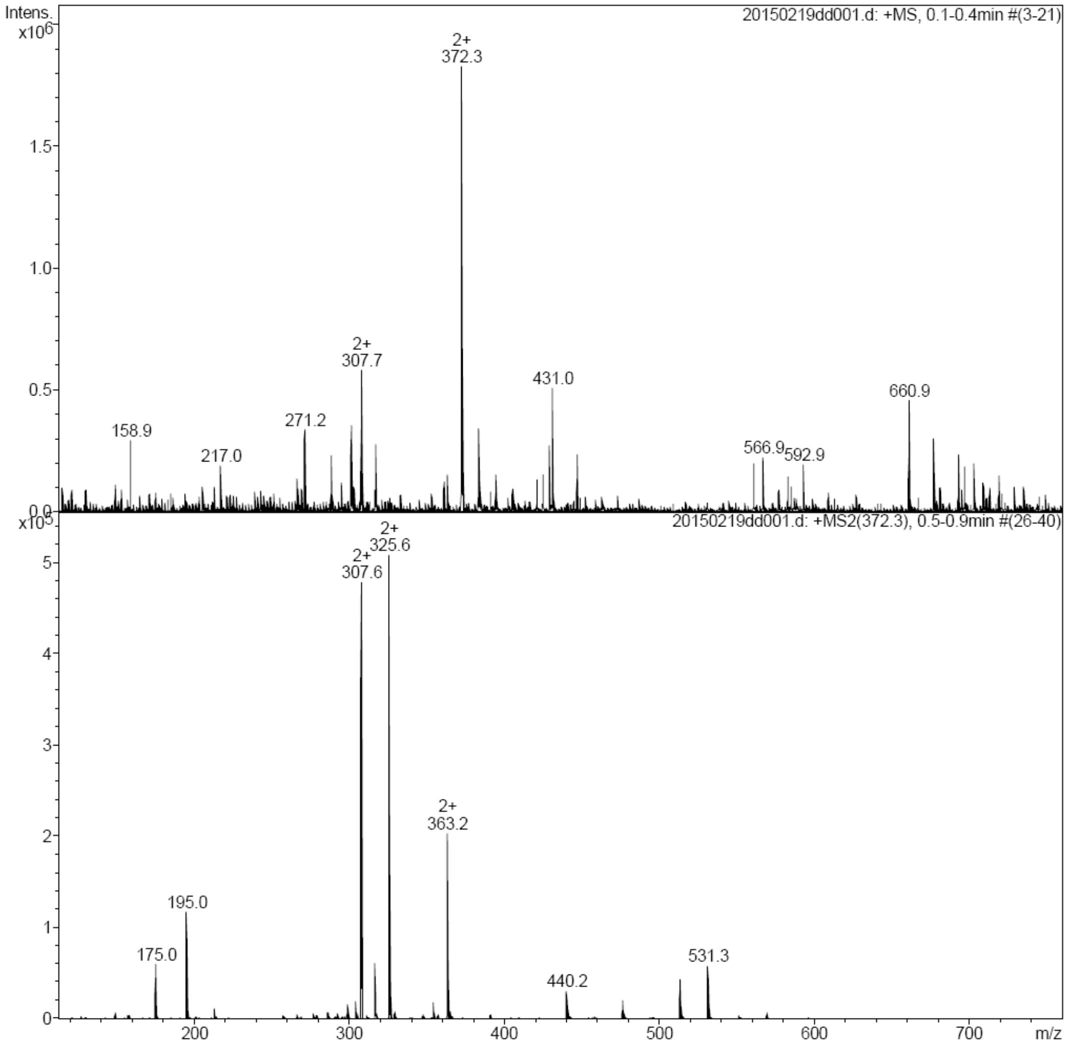

Figure S3. *Cont.*

hexaMC-YR:

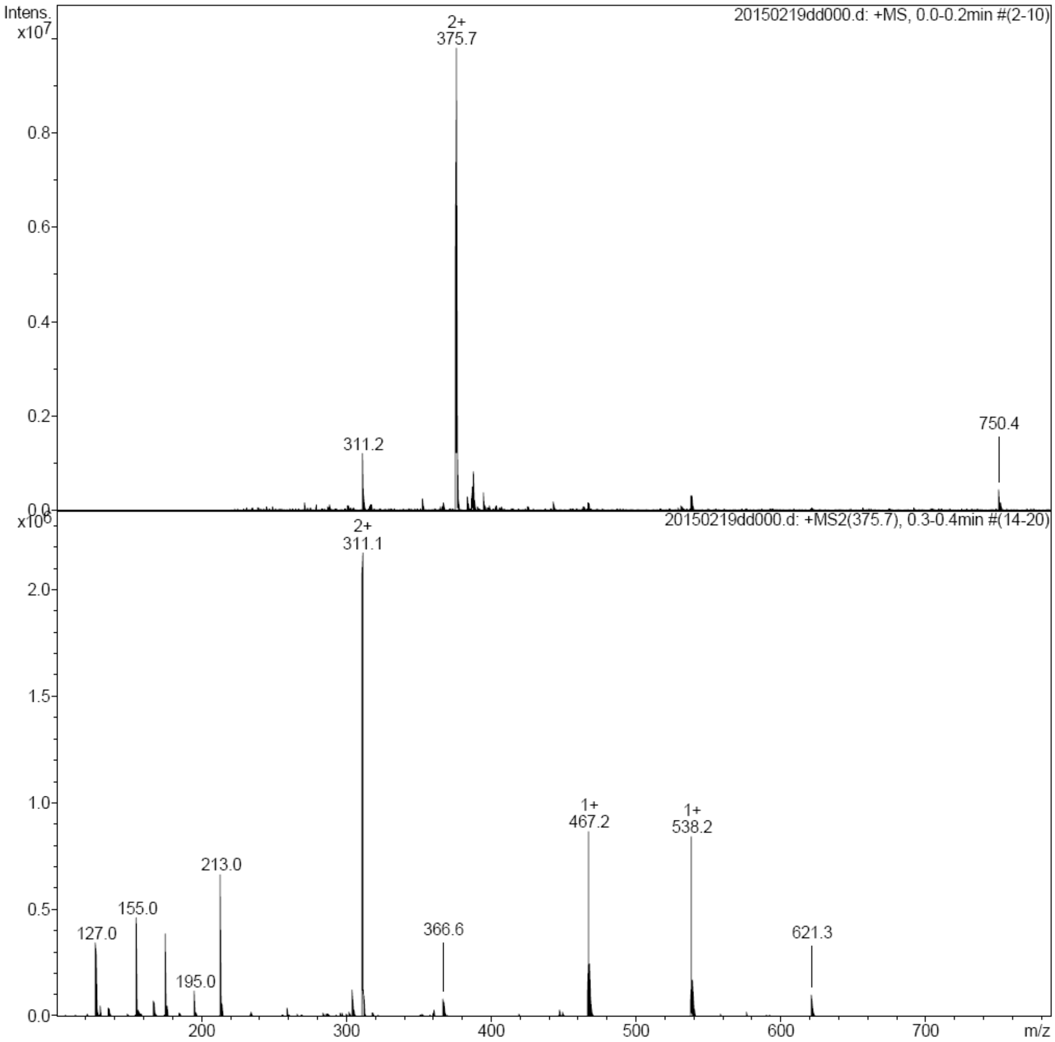

Figure S3. MS^2 of the hexapeptides originated from other MC variants.
